# A dissonance-based intervention for women post roux-en-Y gastric bypass surgery aiming at improving quality of life and physical activity 24 months after surgery: study protocol for a randomized controlled trial

**DOI:** 10.1186/s12893-018-0358-7

**Published:** 2018-05-09

**Authors:** Fanny Sellberg, Sofie Possmark, Ata Ghaderi, Erik Näslund, Mikaela Willmer, Per Tynelius, Anders Thorell, Magnus Sundbom, Joanna Uddén, Eva Szabo, Daniel Berglind

**Affiliations:** 1 Department of Public Health Sciences, Karolinska Institutet, K9, Social Medicin, SE-171 77 Stockholm, Sweden; 2 Department of Clinical Neuroscience, Karolinska Institutet, SE-171 77 Stockholm, Sweden; 3 Division of Clinical Sciences, Danderyd Hospital, Karolinska Institutet, SE-182 88 Stockholm, Sweden; 4 Department of Health and Caring Sciences, University of Gävle, SE-801 76 Gävle, Sweden; 5 Centre for Epidemiology and Community Medicine, Stockholm County Council, Box 45436, SE-104 31 Stockholm, Sweden; 6 Department of Clinical Science at Danderyd Hospital, Karolinska Institutet, SE-116 91 Stockholm, Sweden; 7 Department of Surgery, Ersta Hospital, SE-116 91 Stockholm, Sweden; 8 Department of Surgical Sciences, Uppsala University, SE-751 85 Uppsala, Sweden; 9 Department of Medicine, Karolinska Institutet, SE-141 86 Stockholm, Sweden; 10 Department of Endocrine and Obesity, Capio st Görans Hospital, SE-141 86 Stockholm, Sweden; 11 Department of Surgery, Faculty of Medicine and Health, Örebro University, SE-701 85 Örebro, Sweden

**Keywords:** Bariatric surgery, Roux-en-Y gastric bypass, Dissonance-based, Intervention, RCT, Quality of life, Physical activity

## Abstract

**Background:**

Roux-en-Y gastric bypass (RYGB) surgery is the most common bariatric procedure in Sweden and results in substantial weight loss. Approximately one year post-surgery weight regain for these patient are common, followed by a decrease in health related quality of life (HRQoL) and physical activity (PA). Our aim is to investigate the effects of a dissonance-based intervention on HRQoL, PA and other health-related behaviors in female RYGB patients 24 months after surgery. We are not aware of any previous RCT that has investigated the effects of a similar intervention targeting health behaviors after RYGB.

**Methods:**

The ongoing RCT, the “WELL-GBP”-trial (wellbeing after gastric bypass), is a dissonance-based intervention for female RYGB patients conducted at five hospitals in Sweden. The participants are randomized to either control group receiving usual follow-up care, or to receive an intervention consisting of four group sessions three months post-surgery during which a modified version of the Stice dissonance-based intervention model is used. The sessions are held at the hospitals, and topics discussed are PA, eating behavior, social and intimate relationships. All participants are asked to complete questionnaires measuring HRQoL and other health-related behaviors and wear an accelerometer for seven days before surgery and at six months, one year and two years after surgery. The intention to treat and per protocol analysis will focus on differences between the intervention and control group from pre-surgery assessments to follow-up assessments at 24 months after RYGB. Patients’ baseline characteristics are presented in this protocol paper.

**Discussion:**

A total of 259 RYGB female patients has been enrolled in the “WELL-GBP”-trial, of which 156 women have been randomized to receive the intervention and 103 women to control group. The trial is conducted within a Swedish health care setting where female RYGB patients from diverse geographical areas are represented. Our results may, therefore, be representative for female RYGB patients in the country as a whole. If the intervention is effective, implementation within the Swedish health care system is possible within the near future.

**Trial registration:**

The trial was registered on February 23th 2015 with registration number ISRCTN16417174.

## Background

Obesity with its health-related co-morbidities is a major public health problem world-wide [1]. However, lifestyle interventions, such as dietary restriction and increased physical activity (PA), have limited effect on weight loss and maintenance [2, 3]. On the other hand, Roux-en-Y Gastric Bypass (RYGB) surgery results in marked and sustained weight loss as well as improvements in obesity-related comorbidities, compared with lifestyle interventions [4]. In 2014, laparoscopic RYGB accounted for more than 80% of all approximately 7000 bariatric procedures performed in Sweden, of which 75% were women [5]. With the increased use of laparoscopic sleeve gastrectomy, the proportion of laparoscopic RYGB decreased to 64% of the bariatric procedures performed in Sweden 2016 [6].

Weight regain and reoccurrence of obesity related comorbidities after RYGB are not uncommon [7, 8]. In most cases, this is not caused by surgical issues [9], but rather by difficulties with adaptations to the psychosocial life changes brought about by the procedure [10], or by the patient’s inability to adhere to the prescribed lifestyle recommendations [9]. Patient’s health-related quality of life (HRQoL) and body esteem tend to improve, and is closely associated with weight loss, following RYGB [11, 12]. The improvement in HRQoL typically peaks during the first year after surgery, when the weight loss is most rapid, and then declines as gradual weight regain starts to occur at one to six years after surgery [11]. It is easy to imagine a negative spiral experienced by RYGB patients as their weight loss slows down or weight is regained. Shame and stigmatization lead to increased sedentary behaviors and avoidance of PA, which in its turn might induce even greater problems with body esteem and weight regain. It is therefore important to create efficient preventive measures to avoid this vicious circle.

There is a growing interest in the role of PA and sedentary behavior in achieving optimal weight loss and improving health outcomes after RYGB [13]. Participation in moderate to vigorous intensity PA and reduced sedentary time play an important role in body weight regulation and may contribute to improvements of surgical outcomes after RYGB [14, 15]. In addition, achieving sufficient amounts of PA after surgery is of importance for long-term all-cause and cardiovascular mortality [16] as well as weight maintenance [17].

Although previous research suggests that dysfunctional eating behaviors, issues with body esteem and becoming habitually physically active represents a major challenge for many RYGB patients [10, 12, 18], few interventions to assist patients in meeting these challenges have been conducted. A 2015 systematic review and meta-analysis on interventions before and/or after bariatric surgery stated that the strength of evidence is limited by few trials, low methodological quality and short follow-up duration. The authors concluded that well-designed randomized controlled trials (RCTs) with at least two years follow-up are required. Previous RCTs evaluate weight-loss as the main outcome, with little focus on HRQoL and healthy levels of PA and sedentary behavior. This is of special concern as barriers to engage in PA and exercise, such as feeling too fat, is more common among obese adults [19]. In particular, RYGB patients frequently feel too overweight to exercise, or have fear of exercise-related injuries [15]. Hence, it may be appropriate to aim at reducing sedentary behavior after RYGB surgery, since reduced time spent sedentary may have beneficial effects on health and weight stability in populations with obesity, beyond the effects of light and moderate to vigorous PA (MVPA) [20, 21].

The need for an adequately powered RCT targeting patients undergoing bariatric surgery is justified for several reasons. Firstly, we are not aware of any theory-based counselling intervention for obese female patients after RYGB with appropriate statistical power. Secondly, as RYGB has been the most dominant bariatric procedure performed annually in Sweden during several years [6], it is timely to evaluate the effects of a novel theory-based post-bariatric intervention aimed at facilitating female patients’ psychosocial adjustment to daily life, including HRQoL, eating behavior, mood, body esteem, PA and sedentary behaviors. Such an intervention might also prevent further weight re-gain. Finally, a RCT is needed to evaluate the effects of an intervention, where the attainment of unbiased estimates is crucial and almost impossible to attain by observational studies.

The intervention framework was based on cognitive dissonance theory, which states that psychological distress is created when a person attempts to hold inconsistent sets of cognitions at the same time. People experience dissonance when they are encouraged to act in a way that is contrary to their cognitions, and they are prone to change their cognitions to create consistency [22]. People are also prone to change their future behavior to reduce dissonance [23]. In the current intervention, a modified version of Stice’s dissonance-based prevention model for eating disorders was adapted to RYGB patients. Apart from prevention of eating disorders, the model has been used for smoking cessation, prevention of unhealthy weight gain [24] and for promoting healthy PA behaviors [25].

### Aim

This paper presents the study design and methodology of the “WELL-GBP” intervention (wellbeing after gastric bypass) and describes the baseline characteristics of the participating women. The “WELL-GBP” trial is targeting female RYGB patients in a health care setting to improve HRQoL and health related behaviors after RYGB. The overreaching goal of the current study is to find ways of optimizing the outcome for the RYGB patient, not only in terms of maintained weight loss but also in terms of HRQoL, eating behavior, body esteem, social adjustment, PA and sedentary behavior. The specific aims of the “WELL-GBP” trial are to answer the following questions within a randomized controlled trial:

- What are the effects of a dissonance-based intervention on HRQoL (primary outcome), eating behavior, body esteem and social adjustment of female RYGB patients 24 months after surgery?
- What are the effects of a dissonance-based intervention on objectively measured levels of physical activity and sedentary behavior of female RYGB patients 24 months after surgery?

## Methods and design

This randomized controlled intervention study started in January 2015 in Sweden and is still ongoing: all baseline data has been collected and follow up measurements are going to be collected until end of 2019. The trial operates in three counties (Uppland, Närke and Södermanland counties), where participants are recruited from five hospitals: Uppsala University hospital, Danderyd hospital, St Görans hospital, Örebro University hospital and Ersta hospital. The trial has been approved by the Ethical Review Board Stockholm, Dnr 2013/1847–31/2. The trial has also been registered: ISRCTN16417174.

### Participants

As stated previously, approximately 75% of all bariatric surgery patients in Sweden are women [5]. To avoid lack of power associated with stratification of sex and limited number of male patients, we included only women in the current trial. Participants were recruited at each hospital approximately one to three months before RYGB surgery. The inclusion criteria were severe obesity (BMI ≥ 35 kg/m^2^), being able to understand and speak Swedish and the absence of any serious chronic disease such as stroke or myocardial infarction. All women who fulfilled the inclusion criteria and were accepted for primary RYGB surgery were asked to participate in the study. In general, patients are not eligible for surgery if they are under 18 years old, have not made previous serious attempts to lose weight or alcohol/substance abuse, recent heart disease or stroke or certain kinds of cancer, although the guidelines may to some extent differ between counties.

### Setting and recruitment

**Fig. 1:**
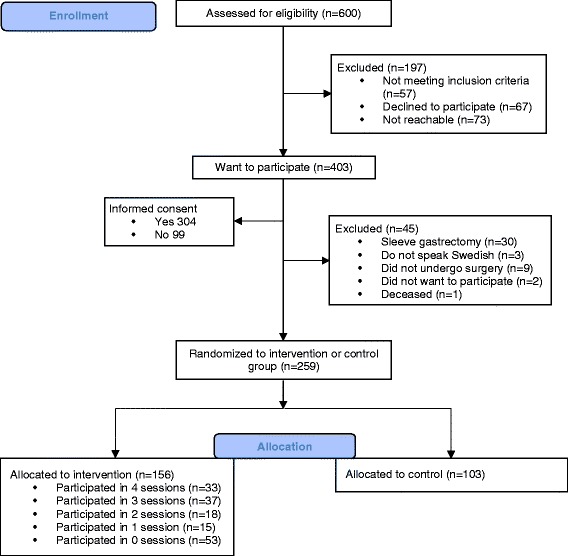
Flowchart of enrollment and randomization

The study is conducted by Karolinska Institutet (KI) in collaboration with the five previously mentioned hospitals for recruitment. Altogether, the five hospitals account for approximately 25% of all bariatric surgery procedures performed in Sweden [6]. The recruitment procedure differed across the hospitals. At Uppsala University hospital and Örebro University hospital, the eligible RYGB patients were sent an information sheet together with a form for declaration of interest to participate in the study approximately three months before surgery. Thereafter the participants filled in the declaration of interest to the hospital, from which it was forwarded to KI. Ersta, Danderyd and St Görans hospitals had group information meetings, led by dietitians and specialized obesity nurses approximately one to three months before surgery. These group meetings occurred weekly and were visited by research staff from the current trial who informed patients about the trial and handed out declarations of interest forms for patients to fill in on-site. For all participants, research staff from KI then contacted the interested patients by telephone for consultation regarding participation in the study (*n* = 600). Participants were excluded if they failed to meet the inclusion criteria (*n* = 57), declined participation (*n* = 67), or if the research staff were unable to reach them by telephone, mail or e-mail (*n* = 73). The eligible participants received an accelerometer (Actigraph GT3X+) and questionnaires together with a form for informed consent, all sent by mail to their homes (*n* = 403). Once the participants sent back baseline assessments they were defined as a participant in the study (*n* = 304) (See Fig. 1). Participants who returned the baseline assessments received the results from their accelerometer measurement together with either a movie ticket voucher or a gift card (value 100 SEK) to promote participant retention. They received the same gift for all follow-up measures. Participant questionnaire were stored in numerical order in a secure and accessible place and manner. Data from questionnaires were entered manually twice by two different researchers to minimize bias. All personal data is handled in a confidential way according to KIs rules and the Swedish law.

Eligible participants who sent back the material (*n* = 304) were excluded from the study if they did not speak Swedish (*n* = 3), did not undergo the planned surgery (*n* = 9), changed their minds about wanting to participate (*n* = 2), died (*n* = 1), or if they underwent a sleeve gastrectomy instead of RYGB (*n* = 30) (see Fig. 1).

Participants were randomized to either intervention group (60%, *n* = 153) or control group (40%, *n* = 103) after surgery. Participants in the intervention group received a telephone call from the research staff approximately two months after RYGB where they obtained more detailed information about the group sessions. The dates for the four sessions were decided by the moderator in an attempt to fit the schedule of all group members. The control group participants were informed by mail.

### Pilot study

The first eight post-surgical patients at Ersta hospital in March and April 2015, were invited to participate in a pilot study (pilots are not included in the flow-chart). The aim was to test the intervention manual to see if it was understandable and acceptable for both the participants and the moderator. The participants provided feedback after each session, and small adjustments to the manual were made. The pilot intervention was conducted in June 2015 and five of the eight participants attended at least one of the sessions.

### Intervention description

The intervention started approximately two to three months after RYGB, and consisted of four group sessions during which a modified version of the Stice dissonance-based intervention model was used [24, 26]. See Table 1 for an overview of the intervention sessions. The sessions were offered once per week during four weeks, at the clinic where the women had surgery. Each session lasted for approximately 1,5 h and preferred group size was approximately five women per group. One of the research staff acted as a moderator and followed an intervention manual. As mentioned above, the intervention was based on the principle of cognitive dissonance theory [27] and the moderator encouraged the participants to talk to each other about how they wanted to act and behave to certain situations in the future, and in that way create the dissonance in relation to how they had been acting before their surgery. The group sessions aimed to provide the participants with strategies for coping with future difficulties with eating and PA behaviors, feelings of shame and other negative emotions, as well as to give them the opportunity to actively engage in thinking about their own and others’ current standpoints and perspectives in these areas. The sessions were not focused on teaching or informing the participants about appropriate eating or PA behaviors, but rather on eliciting active engagement and discussion and to take a stand on preferred ways of handling the discussed difficulties. Each of the four sessions focused on a different aspect of difficulties that might occur following RYGB. The manual for this project was developed by three researchers included in the development of the intervention, of whom two had previous experiences with RYGB patients, and one of whom is professor in clinical psychology.

**Table 1:** Overview of the 4 intervention sessions in groups post RYGB Surgery

Topic for group sessions	Duration (mins)	Time after RYGB (weeks)	Content
1. Physical activity	90	12	-Sedentary behavior and motivation to minimize it -PA, how to avoid failure and make it a routine -How to overcome fears/difficulties with PA for RYGB patients
2. Eating Behaviors	90	13	-How to deal with cravings, powerlessness, feelings of emptiness and shame in relation to eating behavior -Different interpretations of health and appearance -Expectations and relation to food
3. Social relationships	90	14	How to handle different difficult social scenarios after RYGB, for example: -Comments from one’s surroundings about foods -Comments from one’s surroundings about weight loss -Eating at restaurants, buffets, parties
4. Intimate relationships	90	15	-Intimate and sexual relations, sexuality, avoidance and lust -Self-esteem and shame, how to improve it -Summarizing all sessions

The first session included PA and sedentary behavior. The participants were asked to suggest and discuss behaviors that may decrease sedentary behaviors throughout the day. For example, to think about what makes them not carry out planned PA, and to reflect on their own attitudes and approaches to PA. They were also asked to complete a home exercise, which entailed to write down what it meant for them to be physically active, what their long-term goal was in this area, how they were planning to realize their plans, what obstacles they expected to encounter, and how they would cope with them.

The second session addressed eating behavior and problems that are relatively common post- RYGB. The session focused on two (fictional) letters from women who had undergone RYGB two years earlier, and were now facing eating-related problems. The first woman reported extreme cravings for sweet, followed by uncontrolled eating, shame and feelings of loss of control. The second woman described a sense of loss and grief as she could no longer eat the types and amounts of food that she used to, including the foods she used to bake and cook for her family. Additionally, this session included expectations from the participants themselves and their surroundings, and different interpretations of health and appearance. The homework exercise was to write a reply to one of the letters, entailing support, understanding and suggestions for action that marked a clear stance toward the problem and obstacles.

The third session focused on social relations, including family, friends and colleagues. The participants were given the opportunity to reflect on and to role play different scenarios they often experienced themselves. For example, people around them tried to get them to eat foods that are unsuitable after RYGB, gave unhelpful comments or criticisms, or they found themselves at a restaurant with big portions and/or lack of suitable menu options. As a homework exercise each participant wrote a letter to herself to be sent back to her after six months, containing overall goals, mindset and strategies and at least one “lesson learned” from the group sessions.

The fourth session dealt with intimate relations and sexuality. The participants were asked to reflect on and to discuss possible expectations, from themselves and from their partners, as well issues related to lack of a partner, which might arise after surgery. In addition to this, this session also included (fictional) letters. These might have to do with experiencing or not experiencing sexual desire, feelings about one’s own body following major weight loss, and to deal with desired or unwanted sexual attention from others.

During the four sessions, the majority of participants expressed a desire to have more group meetings approximately one year after the surgery, since they felt they wanted to discuss problems and strategies when they had recovered and started to get into their new habits post-surgery. Therefore, a booster session was added and was held approximately one year after surgery. The booster session was a compilation of the four previous sessions. The participants were asked to reflect on the previous topics: PA, eating behavior, social relations and intimate relations and sexuality for approximately ten minutes per topic. Lastly, participants were encouraged to share the experience of receiving the letter six months after they had written it to themselves and to further reflect on experienced problems and possible resolutions after surgery. To the best of our knowledge, none of the participants included in the “WELL-RYGB” Intervention study have suffered any adverse effects so far.

### Measurements

At baseline, approximately one month before RYGB, and at clinical follow-up visits at 6, 12 and 24 months after RYGB, participants will be weighed and have their height measured. The women will, at baseline and at the follow-up visits, be asked to complete a number of questionnaires, sent to their home to fill in and return to KI by mail. The following questionnaires are used:

SF-36 [28] is measuring HRQoL (primary outcome) and is a widely-used instrument which measures health-related quality of life with 36 questions divided in eight dimensions: physical functioning, role limitations due to physical health problems, bodily pain, general health, vitality, social functioning, role limitations due to emotional problems and mental health. We also present data in a summary score for the physical components (PCS) and the mental components (MCS) including a cut off value of ≤42 for the MCS as having a risk for depression. The final scores for the different parts is made into a zero to 100 scale with higher numbers indicating better HRQoL.

Three-Factor Eating Questionnaire (TFEQ) [29] is a 21-item questionnaire measuring dietary restraint, emotional eating and uncontrolled eating. A higher score indicates higher restraint, uncontrolled eating and emotional eating [30, 31].

Body Esteem Scale (BES) [32] is a 23-item questionnaire measuring weight concerns, appearance and attribution. A lower score indicates worse body esteem.

Social Adjustment Scale (SAS) [33, 34] is a 45-item questionnaire measuring satisfaction with one’s social life in the different dimensions: work role, social and leisure activities, relationships with extended family, role as marital partner, parental role and role within the family unit. Lower scores indicates better social adjustment.

Disordered Eating after Bariatric Surgery (DEBS) [35] is a 7-item questionnaire which specifically measures disordered eating after bariatric surgery over the last 28 days. A higher score indicates a higher rate of disordered eating.

Furthermore, women will be asked to wear the validated GT3X+ accelerometer [36, 37] during all waking hours, for seven consecutive days, to objectively measure levels, patterns and intensity of PA and sedentary behavior [38] according to the latest definition, including intensity and posture [39]. Minimal wear time to qualify as a valid measurement is three weekdays and one weekend day with a minimum of ten hours wear time per day.

To be able to compare the intervention to no intervention the control group will receive the usual follow-up care, including weight measures at six and 12 months after RYGB, at the clinic where they had surgery, and like the intervention group, will be asked to complete the questionnaires, to wear the GT3X+ before and six, 12 and 24 months after RYGB. The usual follow-up care differs slightly between hospitals. However, in general it contains consultation with a dietitian about food intake after surgery, and appointments with a nurse and/or the surgeons to measure weight loss, results of laboratory tests and to ensure that no complications have occurred.

### Power calculations

Power calculations showed that 95 participants were needed in each group to attain a statistical power of at least 0.80 (based on HRQoL as the primary outcome), with an expected moderate effect size (Cohen’s d = 50), and alpha set at 0.05. Based on previous similar studies [25, 40], we expect 20% drop-out of patients during two years of follow-up. Thus, a minimum of 240 patients was required, and at final total of 259 participants were recruited and randomized either to the intervention (60%) or to usual care (40%).

### Randomization

Approximately two months after surgery 60% of the participants were block randomized to the intervention group (*n* = 163) and 40% to the control group (*n* = 103). One member of the research staff who was not currently working with the data collection was in charge of the randomization, which was computer generated randomization 60/40. The randomization was divided according to counties (Danderyd, Ersta and St Görans were randomized together, Uppsala and Örebro separately) in blocks of 5 participants and was carried out after date of surgery. Only RYGB participants with informed consent and baseline questionnaire data were randomized.

### Statistical analyses

The statistical analyses will focus on differences between the intervention and the control group from pre-surgery assessment to follow-up assessments at 24 months after RYGB. The women’s changes in weight will be taken into account in the analysis by stratification or adjustment. Primary analysis will be intention to treat analysis, including women who attended at least one session (*n* = 103). We will also conduct per protocol analysis including the women who have attended all four sessions (*n* = 33). We will also perform sensitivity analysis in order to detect any possible dose-response effects such as if the main outcomes will differ depending on if a woman have attended one, two three or all four sessions. Furthermore, individual factors, e.g. patient beliefs about causality of obesity, and contextual factors, e.g. variations in implementation of the intervention between centers for bariatric surgery, might moderate the effect of the intervention on health outcomes. These issues will be analyzed by mixed linear regression models or generalized estimation equations (GEE) to analyze repeated measurements over time. [41].

For the assessments of the baseline characteristics for the intervention and control group presented in this protocol, the analysis have been separated into women randomized to intervention group and women randomized to control. X^2^-tests were performed for categorical variables and t-tests for continuous variables, and because the majority of the continuous variables were not linear distributed, the Kruskal-Wallis test was used.

### Baseline data

This intervention study includes a total of 259 women who have been treated with gastric bypass surgery, of which 156 were randomized to receive a dissonance based intervention consisting of four group sessions, and 103 women to receive usual follow-up care from the clinic where they had surgery. For all tests, there were no statistical significant differences between intervention and control group, which confirms that the randomization was successful. In total, 66% (*n* = 103) of the women in the intervention group attended at least one of the intervention sessions. Baseline characteristics are presented in Table 2. Pre-RYGB measures were: mean age of 44.2 ± 10.5 years (intervention: 43.6 ± 10.7; control: 45.1 ± 10.1), a mean BMI of 40.9 ± 4.7 (intervention: 40.7 ± 4.3; control: 41.2 ± 5.2), a mean weight of 110.9 ± 15.5 kg (intervention: 110.8 ± 14.0; control: 111.0 ± 17.6), and 21% (*n* = 32) in the intervention group and 22% (*n* = 23) in the control group had diabetes type 2.

**Table 2:** Baseline characteristics of women undergoing RYGB Surgery

Characteristics	Intervention (*n* = 156) % (n)/Mean (SD)	Control (*n* = 103) % (n)/Mean (SD)	*p*-value
Age (yrs)	43.6 (10.7)	45.1 (10.1)	0.218
Weight (kg)	110.8 (14.0)	111.0 (17.6)	0.784
Height (cm)	164.9 (6.6)	164.2 (6.7)	0.315
BMI (kg/m^2^)	40.7 (4.3)	41.2 (5.2)	0.712
With diabetes type 2	20.5 (32)	22.3 (23)	0.726
Daily smokers	6.4 (10)	6.8 (7)	0.902
Education			0.535
Primary	12.3 (19)	8.7 (9)	
Secondary	56.1 (87)	54.4 (56)	
Post-secondary	31.6 (49)	36.9 (87)	
Born in Sweden	86.6 (97)	79.7 (59)	0.212

The baseline HRQoL, measured by the SF-36, is shown in Table 3. In general the physical component summary score (PCS) was 42.2 ± 9.6 (intervention: 41.6 ± 9.5; control: 42.9 ± 9.6) and 35% (*n* = 54) in the intervention group and 31% (*n* = 31) in the control group showed an indication for risk of depression (mental component summary score (MCS) ≤42).

**Table 3:** Baseline health-related quality of life, measured by the SF-36, in women undergoing RYGB Surgery

Characteristic	Intervention (*n* = 155^a^) Mean (SD)	Control (*n* = 102^a^) Mean (SD)	*p*-value
Physical Functioning (PF)	58.2 (22.4)	58.8 (24.4)	0.656
Role Physical (RP)	67.7 (28.4)	72.3 (30.1)	0.099
Bodily Pain (BP)	47.9 (28.0)	48.8 (28.8)	0.799
General Health (GH)	50.4 (23.9)	53.1 (22.7)	0.336
Vitality (VT)	37.9 (23.5)	40.1 (35.6)	0.453
Social Functioning (SF)	64.5 (27.9)	64.5 (29.2)	0.922
Role Emotional (RE)	77.2 (28.0)	77.3 (28.5)	0.912
Mental Health (MH)	64.9 (19.5)	64.9 (21.4)	0.770
Physical component summary score (PCS)	41.6 (9.5)	42.9 (9.6)	0.205
Mental component summary score (MCS)	45.8 (11.0)	45.9 (11.3)	0.848
Prevalence of risk of depression (MCS score ≤ 42), % (n)	35.1 (54)	30.7 (31)	0.469

^a^One participant in the intervention group and one participant in the control group did not answer the SF-36 questionnaire

Baseline measurement of the women’s levels of PA, sedentary behavior and wear time are presented in Table 4. The women in the intervention group wore the accelerometer for a mean of 6.6 ± 1.1 days, spent 28.8 ± 17.5 min/day in MVPA and 465.1 ± 98.0 min/day was spent sedentary. For the control group, the women wore the accelerometer for a mean of 6.9 ± 1.4 days, spent 28.8 ± 22.4 min/day in MVPA and were sedentary for 447.0 ± 104.2 min/day.

**Table 4:** Baseline total and intensity-specific levels of accelerometer-measured physical activity (PA) and sedentary behavior (SB) of women undergoing RYGB Surgery

Characteristic	Intervention (*n* = 97^a^) Mean (SD)	Control (*n* = 58^a^) Mean (SD)	*p*-value
Wear time: nr of days (≥10 h/day)	6.6 (1.1)	6.9 (1.4)	0.169
Wear time: hours/day	14.4 (1.2)	14.3 (1.1)	0.369
Total PA (cpm)	576.6 (179.3)	621.2 (230.1)	0.342
Moderate to vigorous PA (min/day)	28.8 (17.4)	28.8 (22.4)	0.579
Light PA (min/day)	369.4 (88.1)	380.2 (78.1)	0.518
SB (min/day)	465.1 (98.0)	447.0 (104.1)	0.399

^a^59 participants in the intervention group and 45 participants in the control group declined to wear an accelerometer or the timing for wearing an accelerometer were too close to their surgery date

## Discussion

The ongoing RCT with a dissonance-based intervention, “WELL-GBP”, targeting female RYGB patients in a health care setting, aims to improve HRQoL and health-related behaviors after RYGB. The intervention is manual-based, free of charge, delivered in health care settings and could be delivered by health-care personnel such as nurses, dietitians, physiotherapists or counsellors, after some practice. We are not aware of any previous randomized controlled trial that has investigated the effects of a similar intervention on HRQoL and following RYGB.

Previous studies using dissonance-based interventions have had positive results on health outcomes and showed greater effect sizes compared to non-dissonance-based interventions or no intervention, regarding disordered eating, body dissatisfaction [24–26], and also increased PA at post-test, but not at 1–2 year follow-up [25, 26]. This trial uses a modified version of Stice’s dissonance-based prevention model and has been adapted to RYGB patients.

The women participating in this trial could be representative of women who undergo RYGB surgery in Sweden, as they are from different geographical areas and are similar in age and BMI at the time of surgery to other RYGB patients in Sweden [42]. They are also comparable in this respect to other bariatric patients from other parts of the world, although they tend to be slightly older and have a higher BMI [43]. A previous study comparing levels of PA pre-bariatric surgery measured by an accelerometer in Swedish women have similar characteristics in the different PA levels as the women in the “WELL-GBP” trial [44]. HRQoL in our sample was similar, although somewhat higher, than the general Swedish bariatric surgery patients [45]. HRQoL, especially PCS score, improves after bariatric surgery and is related to weight loss, gender and age, with lower PCS and MCS scores in women than men, and lower PCS but higher MCS with increased age [45].

The current “WELL-GBP” trial only includes Swedish-speaking women, as resources such as an interpreter at the group sessions were not feasible. This may affect the external validity as the future results will not be representative of women who live in Sweden but don’t master the Swedish language. Also, because this current trial only includes women, any possible intervention effect on men who undergo RYGB will not be possible to predict.

To reduce the problems associated with drop-out, a larger proportion (60%) of the participants were randomized to intervention. Unfortunately, 34% (*n* = 53) of the women in the intervention group did not attend any of the intervention sessions, even though they agreed to participate when invited and had completed the baseline assessments. This may have a negative effect on the upcoming results, but in accordance with the power calculations (see above), a minimum of 95 participants was needed to attain a statistical power of at least 0.80. In this trial 103 women (66%) in the intervention group completed the baseline assessments and attended at least one of the intervention session. Even so, the results may not be representative to the general Swedish women who undergo RYGB surgery.

### Conclusions

The “WELL-GBP” trial is conducted within a Swedish health care setting where female RYGB patients from diverse geographical areas are represented. Our results may, therefore, be representative for female RYGB patients in the country as a whole. If the intervention is effective, implementation within the Swedish health care system is possible within the near future.

## Abbreviations

BMI: Body mass index
HRQoL: Health related quality of life
KI: Karolinska Institutet
MVPA: Moderate to vigorous physical activity
PA: Physical activity
RYGB: Roux-en-Y gastric bypass
WELL-GBP: Wellbeing after gastric bypass
